# Two cases of myeloid sarcoma of the mediastinum

**DOI:** 10.1016/j.radcr.2025.01.035

**Published:** 2025-01-28

**Authors:** Sayo Irie, Akihiro Inoue, Taisuke Nakamura, Yusuke Kobayashi, Tadashi Yamaguchi, Ryo Aoki, Hiroyuki Kamide, Toshiaki Nishii, Zenjiro Sekikawa

**Affiliations:** Department of Radiology, Yokohama City University Medical Center, 4-57 Urafunecho Minami-ku, Yokohama-shi, Kanagawa-ken 232-0024, Japan

**Keywords:** Mediastinal tumor, Myeloid sarcoma, Acute myeloid leukemia, CT-guided biopsy

## Abstract

Myeloid sarcoma is a malignancy characterized by the excessive proliferation of immature myeloid cells or myeloblasts, leading to tumor formation outside the bone marrow. This condition often manifests before or after the onset of acute myeloid leukemia or during a relapse following initial remission. Myeloid sarcoma develops in any organs or parts of the body, but its occurrence in the mediastinum is rare. We report 2 cases of myeloid sarcoma in the mediastinum. The first case was a 63-year-old woman who experienced exertional dyspnea and was found to have a mediastinal mass along with a left pleural effusion on computed tomography (CT). Blood tests revealed abnormal cells, and a diagnosis of acute myeloid leukemia was confirmed by a bone marrow examination. A CT-guided biopsy of the mediastinal mass confirmed myeloid sarcoma. The second case was a 43-year-old man who presented with dyspnea on exertion and was discovered to have a mediastinal mass compressing the trachea and bronchus on CT. Additionally, a soft tissue lesion with bone destruction in his left maxillary sinus was found on CT. His blood tests showed no abnormalities. Since the CT-guided biopsy of the mediastinal mass did not yield a definitive diagnosis, the second biopsy of the maxillary sinus lesion led to the diagnosis of myeloid sarcoma. It is crucial to consider myeloid sarcoma in the differential diagnosis of mediastinal tumors to facilitate early detection and treatment.

## Introduction

Myeloid sarcoma (MS) is a rare extramedullary tumor formed by the proliferation of immature myeloid precursor cells [3]. It represents a unique tissue-based manifestation of acute myeloid leukemia (AML) or transformed myelodysplastic syndromes (MDS), MDS/myeloproliferative neoplasms (MPN), or MPN [2]. Although MS can manifest in various parts of the body, occurrences in the mediastinum are notably rare and infrequently reported [1,3]. In this report, we describe 2 cases of MS in the mediastinum.

## Case report

### Case 1

**Fig. 1:**
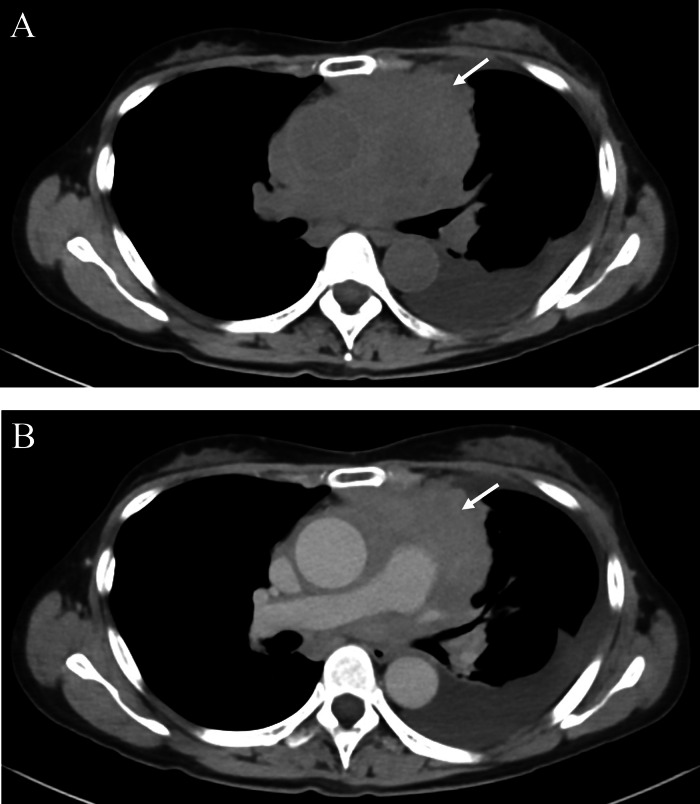
Axial images of plain CT-scan (A), contrast-enhanced CT-scan (B) in the first case. Axial CT images showing poorly defined irregular soft tissue mass (arrow) from the anterior to the middle mediastinum. The mass exhibits homogeneous enhancement (arrow in B). Left pleural effusion is observed.

**Fig. 2:**
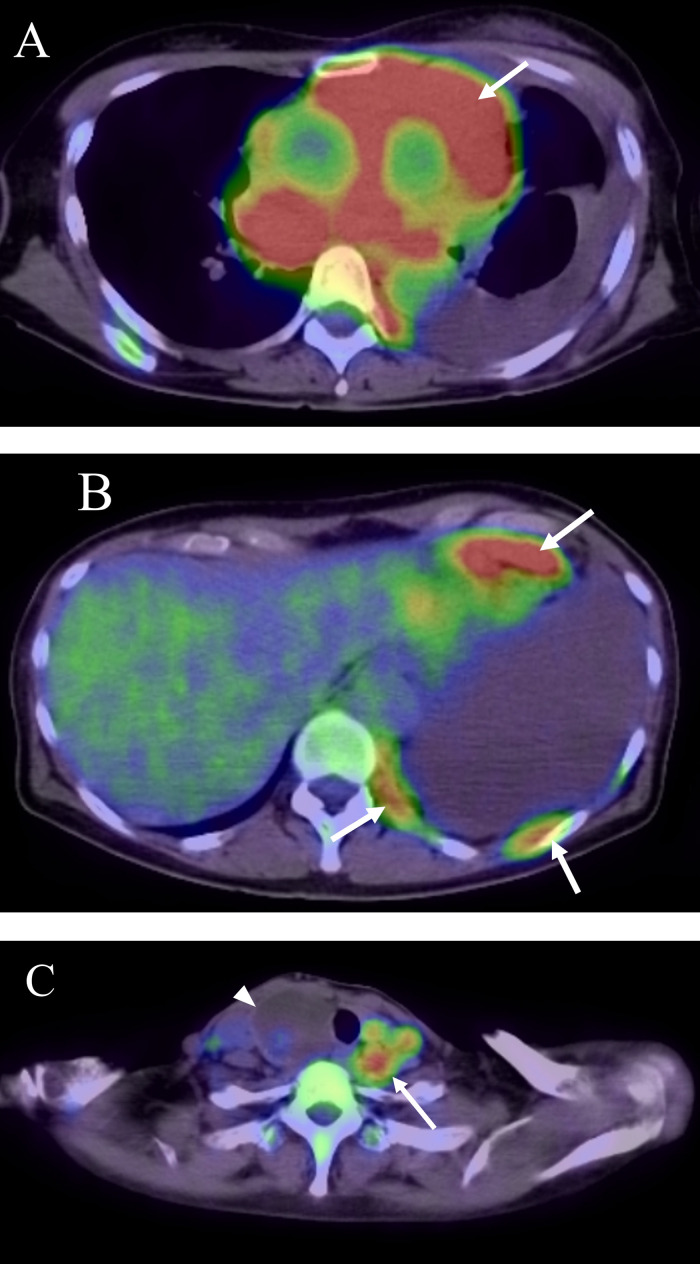
FDG PET-CT scan in the first case. FDG PET-CT scan showing strong uptake (SUV max = 9-12) in the mediastinal mass (arrow in A) along with nodular and linear uptake along the left chest wall (arrow in B). Multiple nodular uptake above the left clavicle (SUV max = 4-6) (arrow in C). Arrow head in C shows a benign thyroid tumor.

A 63-year-old woman with no significant medical history presented with exertional dyspnea. Blood tests showed a white cell count of 7270/μL, with 72.5% of the cells classified as "other," indicating the presence of abnormal cells. A chest computed tomography (CT) scan revealed a poorly defined, irregular soft tissue mediastinal mass measuring approximately 16cm in length. The mass extended from the anterior to the middle mediastinum, extensively involving surrounding structures, including the ascending aorta, pulmonary artery, and superior vena cava. It formed a contiguous mass with the mediastinum and hilar lymph nodes. Homogeneous contrast enhancement was observed in the mediastinal mass. While the mass compressed the left brachiocephalic vein, the vein remained patent. Additionally, the CT scan confirmed the presence of left pleural effusion and atelectasis (Fig. 1). An 18-fluoro-deoxyglucose positron emission tomography (FDG PET-CT) scan revealed strong uptake (SUVmax = 9-12) in the mediastinal mass, along with nodular and linear uptake along the left chest wall. Additionally, multiple nodular uptakes were noted in the para-aortic region and above the left clavicle (SUVmax = 4-6) (Fig. 2). Based on these findings, the differential diagnosis included lymphoma, thymic epithelial tumor, and lung cancer.

To investigate the hematological abnormalities further, a bone marrow biopsy was performed, which revealed AML.

**Fig. 3:**
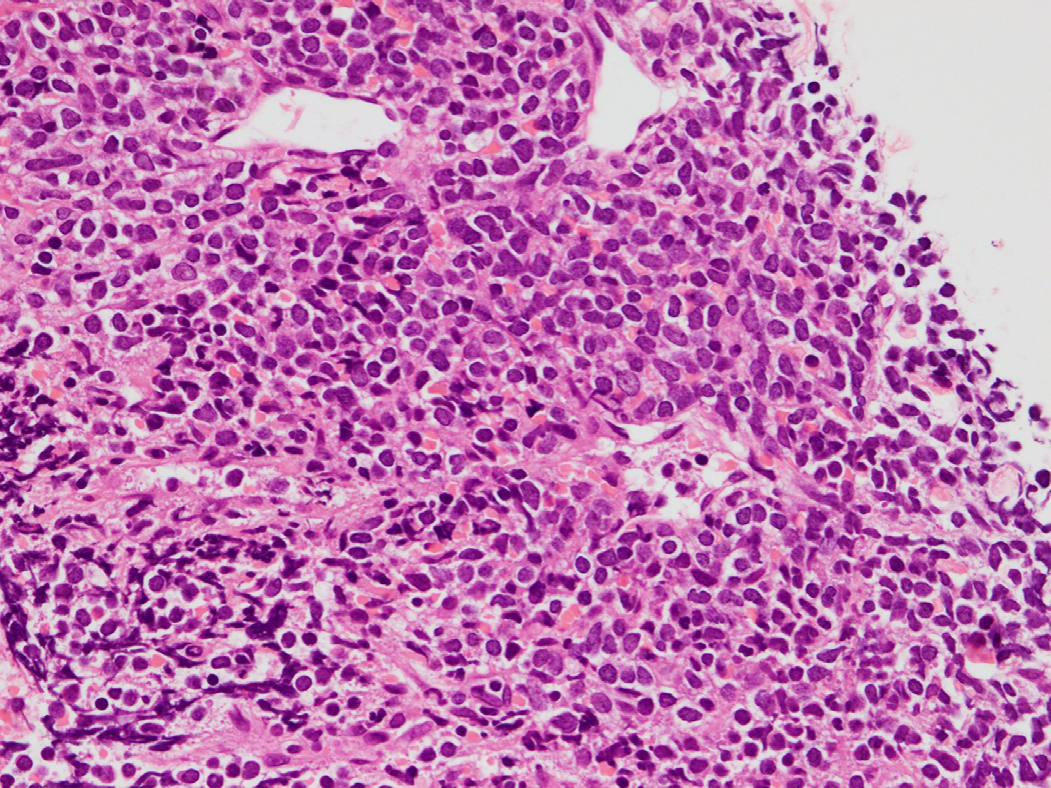
Strongly magnified pathology image of the hematoxylin-eosin staining of the tissue obtained by CT-guided biopsy in the first case. Densely proliferating atypical cells with a high N/C ratio.

Subsequently, a CT-guided biopsy of the anterior mediastinal mass was conducted. Pathological examination showed densely proliferating atypical cells with a high nuclear-cytoplasmic ratio (N/C ratio) (Fig. 3). Immunohistochemical staining was positive for c-kit and CD34, and showed weak positivity for myeloperoxidase (not shown). These findings confirmed the diagnosis of myeloid sarcoma.

**Fig. 4:**
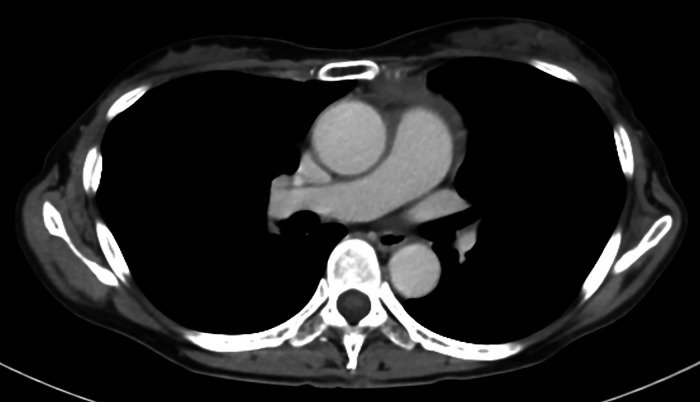
Axial images of contrast-enhanced CT-scan at 3 months after starting treatment in the first case. Axial CT scan image showing shrinkage of the mediastinal mass.

Induction therapy commenced 2 days after the biopsy, and a follow-up chest CT scan confirmed a reduction in the size of the mediastinal tumor (Fig. 4).

### Case 2

**Fig. 5:**
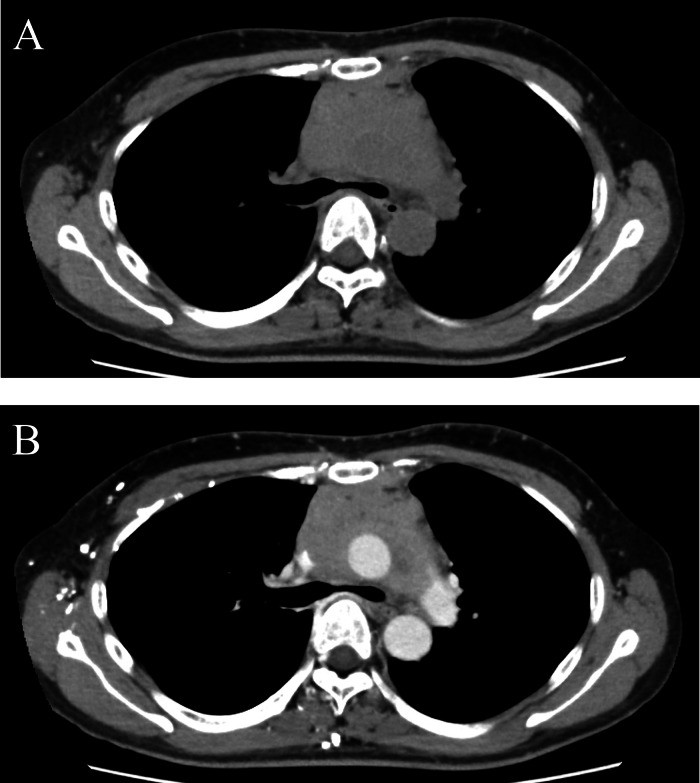
Axial images of plain CT scan(A), contrast-enhanced CT scan(B) in the second case Axial CT scan images showing a poorly defined soft tissue mass in the mediastinum causing left bronchus narrowing. The mass presents homogenous contrast enhancement.

**Fig. 6:**
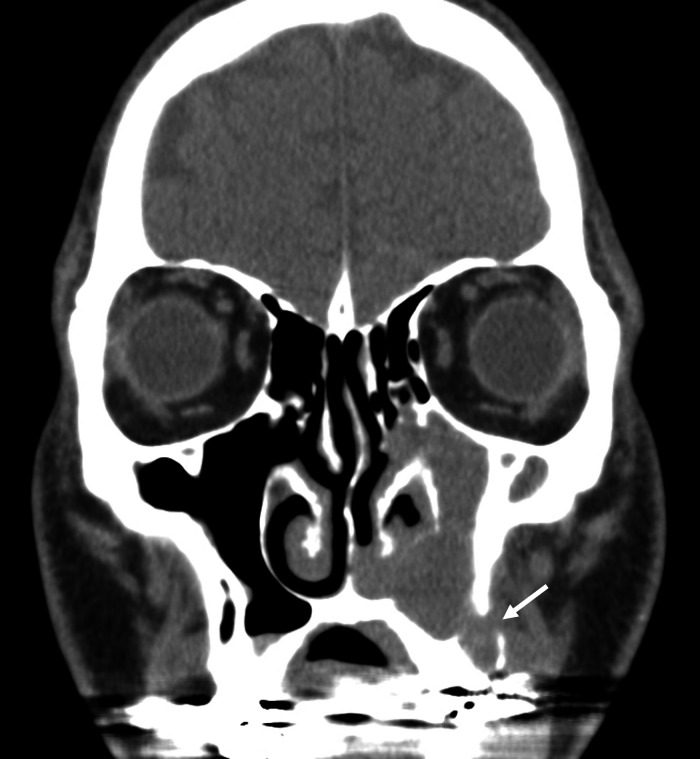
Coronal CT scan of paranasal sinuses in the second case. Coronal CT scan image showing soft tissue lesion in the left maxillary sinus, with thinning and discontinuity of the outer bone wall at the base of the maxillary sinus (arrow).

**Fig. 7:**
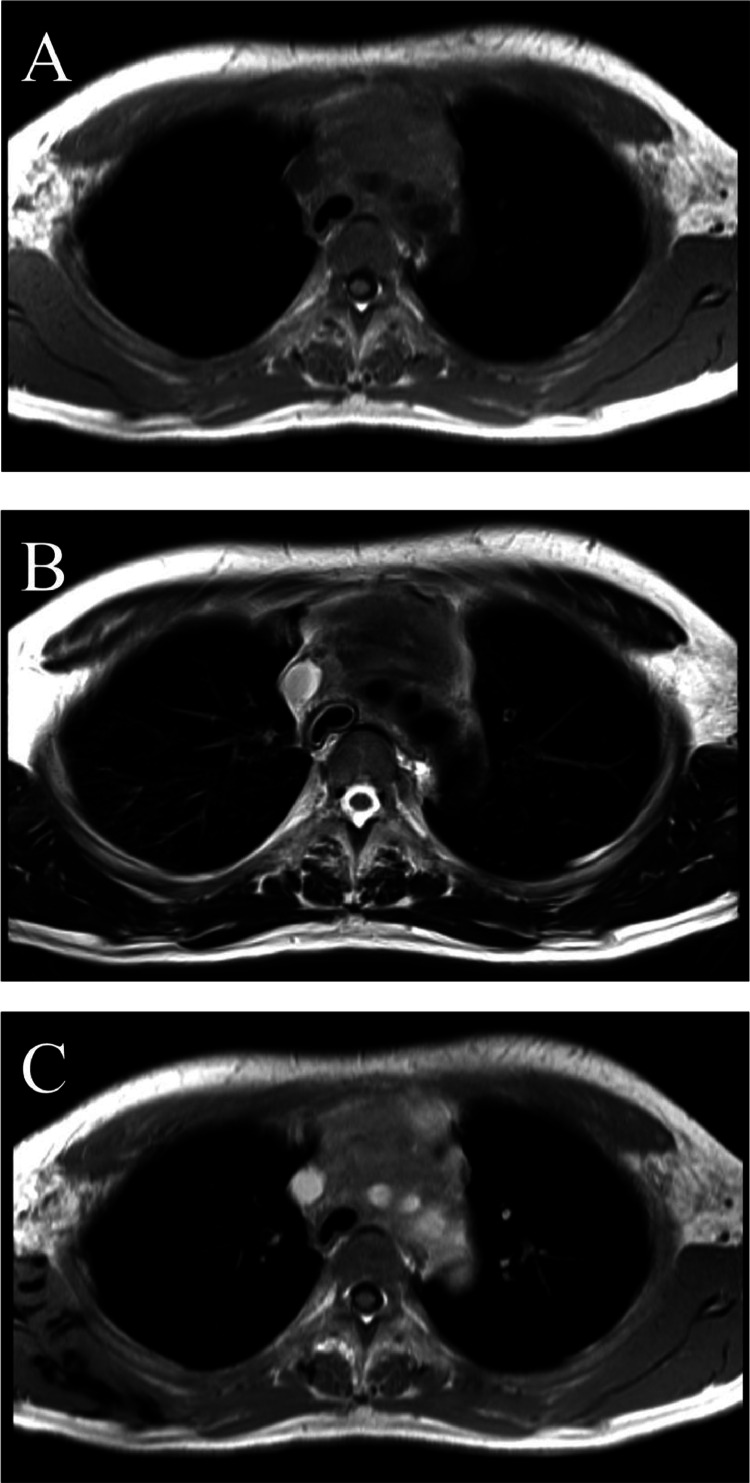
Axial MRI of T1WI(A), T2WI(B), T1WI after injection of Gadolinium (C) in the second case. Axial MRI shows a mediastinum mass with an iso signal on T1-weighted images(A) and a slightly high signal on T2-weighted images(B). Contrast-enhanced MRI showing homogeneous enhancement(C).

**Fig. 8:**
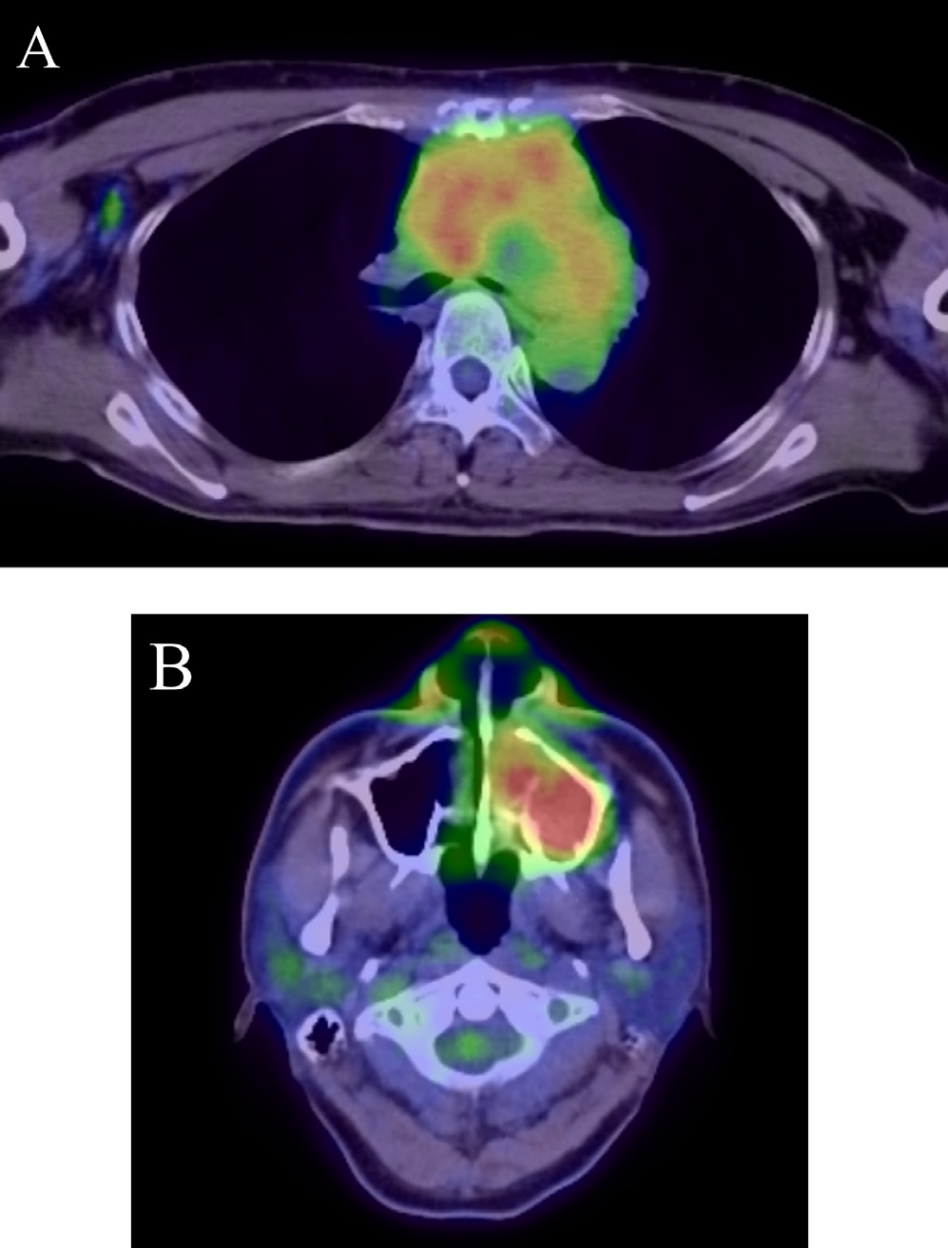
FDG PET-CT scan of chest(A), paranasal sinuses(B) in the second case. FDG PET-CT scan showing abnormal uptake in the mediastinum and right axillary lymph nodes (SUVmax = 2.0-7.5) (A) and in the left maxillary sinus (SUVmax = 8.0)(B).

A 43-year-old man with an untreated anterior chest mass, present for 2 months, experienced exertional dyspnea and sought medical attention. No significant additional medical history was noted, and blood tests showed no abnormalities. A chest CT scan revealed a poorly defined soft tissue mass approximately 10cm in length in the mediastinum. The mass was compressing the trachea and left bronchus, and showing homogeneous contrast enhancement (Fig. 5). Additionally, a soft tissue lesion with thinning and discontinuity of the outer bone wall at its base was observed in the left maxillary sinus (Fig. 6). Magnetic resonance imaging (MRI) indicated that the mediastinal mass had iso-signal intensity on T1-weighted images and a slightly high signal on T2-weighted images. Contrast-enhanced MRI showed homogeneous enhancement of the mass (Fig. 7). An FDG PET-CT scan demonstrated abnormal uptake in the mediastinum surrounding the aortic arch, bilateral internal jugular, bilateral supraclavicular, infraclavicular, right axillary, and subcarinal lymph nodes (SUVmax = 2.0-7.5). Additionally, abnormal uptake was observed in the left maxillary sinus (SUVmax = 8.0) (Fig. 8). Based on these findings, lymphoma, thymic epithelial tumor, and germ cell tumor were considered as potential diagnoses.

A CT-guided biopsy of the anterior mediastinal mass was performed; however, the sample contained no analyzable fragments, and a definitive diagnosis could not be made. This raised suspicion of a hematologic malignancy. Subsequently, a biopsy of the left maxillary sinus lesions was carried out. The pathological examination revealed atypical cells with slightly enlarged nuclei, narrow cytoplasm, relatively uniform chromatin, and small nucleoli. Immunohistochemistry was positive for CD33, CD34, CD79α, and myeloperoxidase, with a Ki-67 positivity rate of 60%. Epstein-Barr virus testing was negative. These findings led to a diagnosis of myeloid sarcoma. It was presumed that the lesions in the mediastinum and left maxillary sinus were caused by the same disease. A bone marrow examination showing 20% blasts confirmed the diagnosis of myeloid sarcoma prior to the onset of AML. Induction therapy was initiated, and initial remission was achieved; however, approximately 1 year later, a relapse occurred, leading to the development of AML. The patient was then transferred to another hospital for continued treatment.

## Discussion

Myeloid sarcoma (MS), also known as granulocytic sarcoma or chloroma, is a solid tumor of extramedullary localization composed of malignant primitive myeloid cells [5]. MS manifests at 4 critical times: ① before the onset of AML, ② after the onset of AML, ③ during an AML relapse following previously induced remission, and ④ during leukemic transformation or progression of other myeloid neoplasms such as MDS, MPN, or chronic myeloid leukemia (CML) [1,3]. Approximately 40% of MS cases occur before the onset of AML [3], and without treatment, around 90% of patients will develop AML within 10.5 to 11 months [6]. The prevalence of MS among AML patients ranges from 2.5% to 9% [5]. MS can develop in various tissues, including the skin, bones, and lymph nodes; however, mediastinal cases are rare, with reports indicating that they account for approximately 2% of cases [1,3]. MS can manifest as a solitary lesion or, as in the second case presented, as multiple lesions [3,5,7].

While imaging studies are valuable for detecting lesions, their findings are often nonspecific, making pathological examination essential for a definitive diagnosis. To diagnose MS in the mediastinum, a CT-guided needle biopsy or surgical biopsy is necessary.

On a plain CT scan, MS typically appears as a mass with a density similar to muscle tissue and exhibits moderate enhancement, which can be either homogeneous or heterogeneous [7].

Various MRI signal changes are observed in MS. Signal intensity usually matches that of skeletal muscles on T1-weighted images and is slightly higher on T2-weighted images [7]. Diffusion-weighted imaging often shows high signal intensity accompanied by decreased apparent diffusion coefficient (ADC) values, with the mean ADC reported to be approximately 0.76 ± 0.19 × 10^− 3^ mm^2^/s [7].

In PET-CT, the lesions in MS generally display moderate to intense FDG uptake (SUVmax, 2.6-9.7), making this examination effective for diagnosing the condition [7,8].

MS is frequently associated with subsequent leukemogenesis following either surgical resection or radiotherapy alone, necessitating chemotherapy similar to that used for treating AML [1,3]. If MS is left untreated, approximately 90% of patients will develop AML within 1 year [6], while the overall survival rate after AML onset is poor, ranging from 6 to 14 months [4].

The differential diagnosis of MS extending from the anterior to middle mediastinum includes lymphoma, thymic epithelial tumors, and germ cell tumors. Distinguishing MS from lymphoma is particularly challenging, as both may manifest as solitary or multifocal lesions. Certain features often associated with lymphoma, such as the penetrating vessel sign or the presence of pleural effusion, may also be observed in MS [[9], [10], [11]]. An elevated serum level of soluble interleukin-2 receptor (sIL-2R) should raise suspicion of lymphoma.

Thymic epithelial tumors frequently present as anterior mediastinal masses and must be considered in the differential diagnosis. Imaging features suggestive of thymic epithelial tumors include calcifications, partial encapsulation or septation, and T2-weighted hypointense areas indicative of fibrosis. A washout pattern on contrast-enhanced imaging is particularly suggestive of thymic epithelial tumors, especially low-risk thymomas. In contrast, lymphomas or malignant germ cell tumors do not exhibit a washout pattern and typically show either persistent or plateau enhancement [9].

Malignant germ cell tumors share imaging findings similar to those of MS. In young males, elevated levels of alpha-fetoprotein or human chorionic gonadotropin strongly suggest malignant germ cell tumors [9].

The imaging findings of MS are nonspecific, making a diagnosis based solely on imaging studies difficult. When a hematological disorder such as AML, CML, or MDS has been established, it may be reasonable to consider MS in the differential diagnosis of a mediastinal mass. However, this can be challenging in the absence of a hematological disorder. Given the high risk of developing AML in patients with MS, the presence of a mediastinal mass necessitates a definitive diagnosis through biopsy.

## Conclusion

MS is a rare condition that can precede AML, highlighting the need for early diagnosis and treatment. However, imaging findings for MS are nonspecific, making it challenging to distinguish from other conditions. We presented 2 cases of MS occurring in the mediastinum. Although this is an uncommon location for MS, considering this condition when differentiating mediastinal tumors to establish an early diagnosis is crucial.

## Patient consent

Informed written consent was obtained from all patients for the publication of the case reports and all imaging studies.
